# A35 DNA HYPOMETHYLATION INHIBITS TUFT CELL-DERIVED COLITIS-ASSOCIATED CANCER

**DOI:** 10.1093/jcag/gwab049.034

**Published:** 2022-02-21

**Authors:** F Larsen, H J Good, A E Shin, L Zhang, S Asfaha

**Affiliations:** 1 Western University Department of Medicine, London, ON, Canada; 2 Western University Department of Pathology and Laboratory Medicine, London, ON, Canada

## Abstract

**Background:**

Colorectal cancer is the second leading cause of cancer death in Canada. A major risk factor for the development of colorectal cancer is chronic inflammation leading to colitis-associated cancer (CAC). We previously described a CAC mouse mode in which tumors arise from DCLK1+ tuft cells following loss of the tumor suppressor adenomatous polyposis coli (APC) and induction of colitis. Interestingly, both colitis and CAC display epigenetic changes that modulate gene expression. Specifically, DNA methylation is altered in colitis, but its role in colonic tumorigenesis is not known. We hypothesize that inhibition of DNA methylation in DCLK1+ tuft cells reduces colonic tumorigenesis.

**Aims:**

In this study, we aim to investigate the role of DNA methylation in CAC by inhibiting DNA methylation by genetic and pharmacologic means.

**Methods:**

We crossed our Dclk1-CreERT2/Apc^f/f^ mice to DNMT1^f/f^ mice to delete the DNA methyltransferase DNMT1 in DCLK1+ tuft cells. We induced CAC in Dclk1/Apc^f/f^ and Dclk1/Apc^f/f^/DNMT1^f/f^ mice by administering three doses of tamoxifen followed by 2.5% dextran sodium sulfate (DSS) for five days. Fourteen weeks later we assessed colonic tumor number and size. Lineage tracing of Dclk1+ cells was also examined in colonic tissues from all mice. In a separate cohort of Dclk1/Apc^f/f^ mice, we induced colitis and treated the mice with six doses of the DNA de-methylating drug 5-AZA-2’-deoxycytidine (5-AZA) or vehicle. Ki67 immunostaining was additionally performed to assess cellular proliferation in the colon.

**Results:**

Deletion of DNMT1 in DCLK1+ cells significantly inhibited the number and size of colonic tumors. Treatment of mice with 5-AZA similarly reduced the overall number of mice with tumors, as well as, the number and size of tumors per mouse. Interestingly, 5-AZA treatment was associated with reduced colonic proliferation as assessed by fewer Ki67+ cells, and quiescent DCLK1+ cells that did not lineage trace. Furthermore, deletion of DNMT1 or treatment with 5-AZA reduced the number of lineage tracing events detected upon exposure to low DSS.

**Conclusions:**

Our findings demonstrate that loss of Dnmt1 or 5-AZA treatment reduces CAC formation. Furthermore, 5-AZA appears to exert its anti-tumor effects by reducing proliferation and preventing tuft cell stemness. Our data demonstrates that altering DNA methylation plays an important role in CAC.

**Funding Agencies:**

CIHR

